# Disopyramide Therapy in Cats with Obstructive Hypertrophic Cardiomyopathy Non-Responsive to Carvedilol

**DOI:** 10.3390/vetsci12100999

**Published:** 2025-10-16

**Authors:** Shuji Satomi, Ryohei Suzuki, Yunosuke Yuchi, Haruka Kanno, Miyuki Nomura, Takahiro Teshima, Hirotaka Matsumoto

**Affiliations:** Laboratory of Veterinary Internal Medicine, School of Veterinary Medicine, Faculty of Veterinary Science, Nippon Veterinary and Life Science University, Tokyo 180-8602, Japan; jetlog21117@yahoo.co.jp (S.S.); y.0301.yunosuke@gmail.com (Y.Y.); teshima63@nvlu.ac.jp (T.T.); matsumoto@nvlu.ac.jp (H.M.)

**Keywords:** cardiac biomarker, echocardiography, feline, heart, left ventricular outflow obstruction, myocardial function, myocardial injury, speckle tracking, strain, systolic anterior motion

## Abstract

**Simple Summary:**

Hypertrophic cardiomyopathy is the most common form of cardiomyopathy in cats. When accompanied by left ventricular outflow tract obstruction, the condition is typically managed with medical therapy in humans. Beta-blockers are generally the first-line treatment, but some cases do not respond to them. In such instances, the antiarrhythmic drug disopyramide is considered as an adjunctive therapy. However, its use in cats has only been documented in a case report. This study aimed to evaluate the efficacy of disopyramide as co-therapy in cats with hypertrophic cardiomyopathy and left ventricular outflow tract obstruction that are refractory to beta-blockers. In this study, adding disopyramide resulted in the alleviation of left ventricular outflow tract obstruction and improvement in myocardial function. Furthermore, disopyramide reduced cardiac troponin I levels, a cardiac biomarker indicating myocardial injury. The results suggest that disopyramide may be a potential therapeutic option for feline obstructive HCM, as no significant side effects were observed.

**Abstract:**

Hypertrophic cardiomyopathy (HCM) is the most common cardiomyopathy in cats and is classified as obstructive (HOCM) or non-obstructive based on anatomical differences in the left ventricular outflow tract (LVOT). In severe obstructive cases, while beta-blockers are the recommended initial treatment in humans, some patients exhibit treatment resistance. For these cases, the addition of the antiarrhythmic agent disopyramide is common. However, its use in cats has only been documented in a case report. In this study, the use of disopyramide resulted in a significant reduction in the LVOT velocity and cardiac troponin I levels. Additionally, no significant adverse effects were observed. These findings suggest that disopyramide could be a potential therapeutic option for the treatment in cats with HOCM.

## 1. Introduction

Hypertrophic cardiomyopathy (HCM) in cats is pathologically characterized by myocardial hypertrophy, disorganized myocardial fiber arrangement, and fibrosis, leading to diastolic dysfunction and subsequently heart failure [1]. When HCM is accompanied by left ventricular (LV) outflow tract obstruction (LVOTO), mainly due to systolic anterior motion of the mitral valve (SAM), it is defined as obstructive hypertrophic cardiomyopathy (HOCM) [2,3]. In humans, obstruction in the LVOT causes symptoms such as chest pain and exertional fatigue [4,5]. Furthermore, the presence of LVOTO is considered a poor prognostic factor, associated with arrhythmias, heart failure, and sudden cardiac-related death [3].

As noted in the American College of Veterinary Internal Medicine consensus guideline for feline cardiomyopathy, treatment intervention should be considered if cats with HCM have severe LVOTO [6]. In addition, in humans with HOCM, when the resting LVOT pressure gradient exceeds 30 mmHg, medical intervention is considered to reduce the intraventricular pressure gradient [4,5]. Beta-blockers are the first line of treatment, and the dosage is increased until symptoms are improved unless adverse effects occur [7,8,9]. Conversely, although no established guidelines exist for the treatment of HOCM in cats, the use of atenolol, a beta-blocker, may be considered in cases of significant LVOTO [6]. However, resistance to beta-blocker treatment in cats with HOCM was reported [10,11]. Therefore, additional treatment options to relieve LVOTO are considered necessary.

In humans, the concurrent administration of disopyramide, a class Ia antiarrhythmic agent, is recommended in cases demonstrating resistance to beta-blocker therapy [4,5]. Disopyramide exerts a negative inotropic effect through sodium channel inhibition, which has been reported to alleviate LVOTO [12,13,14]. While combination therapy with beta-blockers and disopyramide is commonly used in humans, to the best of our knowledge, only one case report described the use of beta-blockers and disopyramide in cats with HOCM [15]. Thus, the efficacy and side effects of disopyramide in cats remain unclear. Therefore, this study aimed to investigate the efficacy and safety of disopyramide in cats with HOCM that were non-responsive to beta-blocker therapy and hypothesized that adjunctive disopyramide therapy would also alleviate LVOTO in cats.

## 2. Materials and Methods

This study was prospective and observational. All study procedures were approved by the Institutional Animal Care and Use Committee of our university (Approval No. R2-4). Written informed consent for the use of study data was obtained in advance from the owners of each cat.

### 2.1. Animals

This study recruited client-owned cats diagnosed with HOCM at our university between April 2023 and July 2024. Of these, those without improvement in LVOTO with carvedilol, a beta-blocker, treatment were included in this study. All cats underwent complete physical examination, electrocardiogram, thoracic radiography, non-invasive blood pressure measurement (oscillometric method), and echocardiography. The diagnostic criteria for HCM were maximal LV diastolic wall thickness at end-diastole of 6 mm or more from right parasternal long-axis or short-axis views on echocardiography. Cats with HCM that also exhibited LVOT velocity (LVOTV) of ≥3.5 m/s from the left apical five-chamber view on continuous-wave Doppler were diagnosed with HOCM [16]. Exclusion criteria included systolic blood pressure >160 mmHg, hyperthyroidism, dehydration, and other cardiac diseases that could cause myocardial hypertrophy.

In this study, cats in whom LVOT did not reach <3.5 m/s after carvedilol administration for at least 30 days were defined as non-responsive to carvedilol treatment and subsequently received co-disopyramide therapy. Given the lack of clearly defined disopyramide dosages for cats, the dosage regimen from a prior case report was referenced [15]. In this study, disopyramide was initiated at approximately 5.0 mg/kg q 12h. After at least two weeks of disopyramide administration, if LVOTV did not reach the target of <3.5 m/s and no issues with side effects and medication compliance were observed, the dose was increased up to double this amount. The dosage of carvedilol was not altered after the initiation of disopyramide. The post evaluation was conducted at least two weeks after initiating disopyramide administration or after increasing the dosage of disopyramide.

### 2.2. Standard Echocardiography

Conventional two-dimensional and Doppler echocardiography was performed by a single investigator (R.S) using an echocardiographic system (Vivid E95 Ultra Edition; GE Healthcare, Tokyo, Japan) and a 12 MHz transducer. During the examination, a simultaneous electrocardiogram (ECG) (lead II) was recorded and displayed on images. All data were obtained for at least five consecutive cardiac cycles. All non-sedated cats were manually restrained in the right and left lateral recumbency. The recorded images were analyzed by a single trained observer (H.K.) using an offline workstation (EchoPAC ver.201; GE Healthcare, Tokyo, Japan).

The left atrial to aortic root ratio (LA/Ao) was measured in the right parasternal short-axis view using the B-mode method. The end-diastolic interventricular septum thickness (IVSd), end-diastolic LV posterior thickness (LVPWd), end-diastolic LV internal dimension (LVIDd), end-systolic LV internal dimension (LVIDs), and fractional shortening (FS) were measured in the right parasternal short-axis view at the level of chordal tendineae using the B-mode method. The relative wall thickness (RWT) was calculated following the formula [17]:

RWT = (IVSd + LVPWd)/LVIDd.(1)

In the left apical four-chamber view, the peak velocity of the early diastolic wave (E-wave) and peak velocity of the late diastolic wave (A-wave) were measured using the pulsed wave Doppler method. The E/A velocity ratio was then calculated. If the E and A waves were fused, the values were excluded. The LVOTV was measured using the continuous-wave Doppler method in the left apical five-chamber view. At the most stable heart rate (HR), the minimum LVOTV value was defined as LVOTV_rest_, whereas the maximum value was defined as LVOTV_excited_ [4,6]. No specific interventions have been applied to obtain the LVOTV_excited_, as in humans with HOCM. For all analyses, the mean values of three consecutive cardiac cycles in sinus rhythm from high-quality images were used.

### 2.3. Two-Dimensional Speckle Tracking Echocardiography

Speckle-tracking echocardiography (STE) is an imaging analysis technique that enables automatic tracking of myocardial motion by performing pattern-matching on images acquired through B-mode echocardiography, whose clinical utility has been demonstrated in both humans and cats [18,19,20]. In the present study, LV circumferential strain was assessed using right parasternal short-axis views at the level of the chordae tendineae, while longitudinal strain was evaluated using left apical four-chamber views. Both longitudinal and circumferential strains were measured separately by myocardial layer: the endocardial, epicardial, and whole layers of the LV. Furthermore, the endocardial-to-epicardial strain ratio (End/Epi), an index reflecting compensatory mechanisms of the endocardial layer, was calculated [21,22]. The mean values of the measurements from three consecutive cardiac cycles obtained from high-quality images were used for all statistical analyses.

### 2.4. Cardiac Troponin I Measurement

For the measurement of cardiac troponin I (cTnI), venous blood samples were collected on the same day as the echocardiographic examination. Serum was separated from whole blood by centrifugation at 1700× *g* for 15 min at 4 °C, and the obtained samples were stored at −80 °C until analysis. The chemiluminescence immunoassay method used to measure cTnI was outsourced (FUJIFILM VET Systems Co., Ltd., Tokyo, Japan). The reference range was set at 0.121 ng/mL or lower.

### 2.5. Electrocardiography

A 20 S ECG examination was performed with the cat restrained in the right lateral recumbency, and data were recorded at a paper speed of 50 mm/s. From these recordings, the QT interval obtained was measured from the onset of the Q wave to the end of the T wave. The QT interval was manually measured over 10 consecutive cardiac cycles, and the average value was used for statistical analysis. Considering the variety of heart rates, the QT interval was normalized with the following formula [23,24]:

QTc = (QT interval [ms])/(HR [bpm])^1/3^ (2)

### 2.6. Statistical Analysis

Statistical analyses were performed using a commercially available software (R 2.8.1; https://www.r-project.org/ [accessed on 22 May 2025]). The Shapiro–Wilk test was used to assess the normality of data distribution. Two paired groups were compared using the paired *t*-test for normally distributed data and the Wilcoxon signed-rank test for non-normally distributed data. The correlation between LVOT velocity and cTnI concentration was evaluated using Spearman’s rank correlation coefficient. All variables were presented as median and interquartile range, and a *p*-value of <0.05 was considered statistically significant.

## 3. Results

A total of 53 cats with HOCM met the inclusion criteria for this study. Among them, 43 cats received monotherapy with carvedilol, of which 36 were excluded due to improvement in LVOTV (decreased to <3.5 m/s). The remaining seven cats started combination therapy with disopyramide. For four cats, since the initial dose of disopyramide showed poor therapeutic response, the dose was increased. One was withdrawn from treatment due to decreased appetite attributed to taste. Finally, six cats were included in the present study.

### 3.1. Clinical Profiles

**Figure 1:**
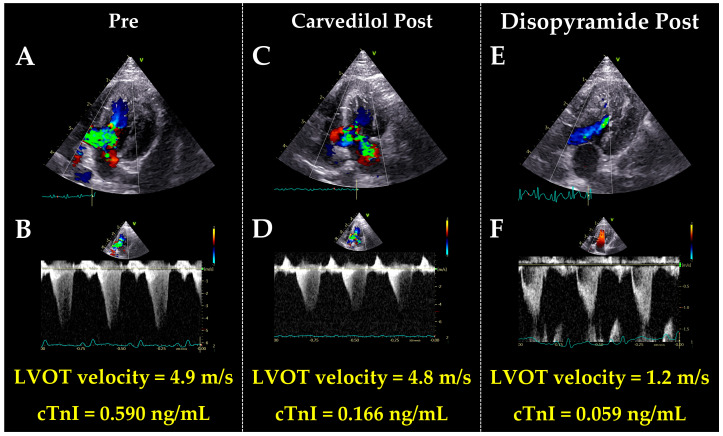
Echocardiographic findings in a representative case showing a decrease in both the peak left ventricular outflow tract (LVOT) velocity and cardiac troponin I (cTnI) following disopyramide administration. Before treatment echocardiograms showed systolic anterior motion of the mitral valve and severe LVOT obstruction (**A**,**B**). Additionally, cTnI level was elevated. After treatment with carvedilol, LVOT obstruction persisted, and cTnI remained elevated (**C**,**D**). After the addition of disopyramide, LVOT obstruction improved, and cTnI returned to the normal range (**E**,**F**).

**Figure 2:**
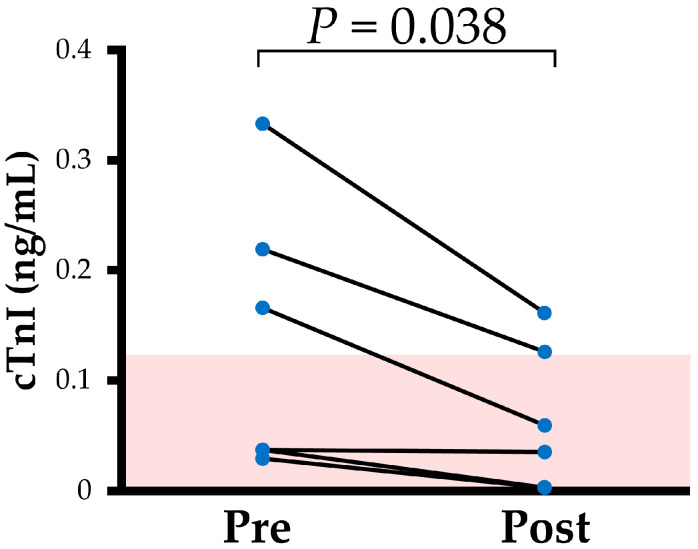
Line graphs of cardiac troponin I for Pre and Post disopyramide treatment in cats with obstructive hypertrophic cardiomyopathy. Blue dots show the individual data points. Pink bar shows the reference range of healthy cats (<0.121 ng/mL). cTnI: cardiac troponin I.

In all cats included in this study, clinical severities of HCM were ACVIM Stage B1 both pre- and post-treatment. Additionally, no changes were observed in body weight, systolic blood pressure, heart rate, QT, and QTc (Table 1 and Figure 1). The cTnI levels decreased after disopyramide treatment (Figure 1 and Figure 2).

### 3.2. Echocardiography

**Figure 3:**
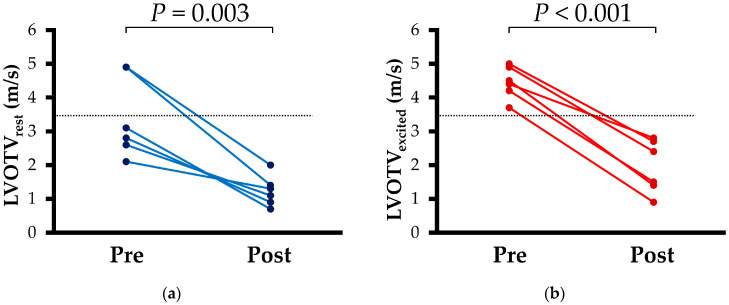
Line graphs of left ventricular outflow tract velocity (LVOTV) for Pre and Post disopyramide treatment in cats with obstructive hypertrophic cardiomyopathy: (**a**) LVOTV_rest_, (**b**) LVOTV_excited_. Blue dots and red dots show individual data points of LVOTV_rest_ and LVOTV_excited_, respectively. Dot line shows the treatment goal of left ventricular outflow tract velocity (<3.5 m/s) in cats with obstructive hypertrophic cardiomyopathy in this study.

Results of echocardiographic measurements are summarized in Table 2 and Table 3. Both LVOTV_rest_ and LVOTV_excited_ decreased post-disopyramide treatment compared to those of pre-treatment (Figure 3). Furthermore, IVSd decreased with disopyramide treatment. Regarding STE analysis, no changes were observed in longitudinal or circumferential strain before and after disopyramide administration.

### 3.3. Correlation Analyses

Results of the correlation analysis are shown in Table 4. cTnI concentrations showed a positive correlation with both LVOTV_rest_ and LVOTV_excited_. No correlation was observed among myocardial function, cTnI, and LVOTV.

## 4. Discussion

In cats with HOCM non-responsive to carvedilol, the addition of disopyramide improved LVOTO. In humans, it is reported to achieve long-term LVOTO relief in two-thirds of cases with the concomitant use of disopyramide [14]. In this study, LVOTO was relieved in all cats except for one case which was withdrawn from the study due to poor medication compliance related to the taste of disopyramide. Additionally, this alleviation of LVOTO was achieved without worsening myocardial function as assessed by STE and was accompanied by a reduction in myocardial cell injury based on cTnI levels. These findings may suggest the potential efficacy and safety of disopyramide in felines with HOCM.

The SAM occurs when the anterior leaflet of the mitral valve displaces towards the interventricular septum, narrowing the LVOT orifice. This increases intraventricular pressure, leading to high-velocity blood flow through the LVOT [25,26,27,28,29]. Disopyramide reduces the rate of the action potential rise, which is believed to delay the apposition of the mitral valve anterior leaflet and the interventricular septum, thereby suppressing SAM and leading to the alleviation of LVOTO [30,31]. Although the specific mechanism of SAM was not investigated in this study, it is presumed that the LVOTO alleviation was achieved based on the effect observed in HOCM humans treated with disopyramide.

Myocardial function, as assessed by two-dimensional (2D)-STE, did not excessively worsen with carvedilol and disopyramide combination therapy. In both humans and cats with HOCM, the negative chronotropic and inotropic effects of beta-blockers improve left ventricular filling through prolongation of diastolic time, thereby enhancing myocardial function and alleviating LVOTO [32,33,34]. Conversely, disopyramide reduces intracellular sodium influx, which secondarily decreases intracellular calcium via the Na/Ca exchanger. This mechanism raises concerns that the administration of disopyramide in HCM non-responsive to beta-blockers could further depress myocardial contractility. In humans, the combination of beta-blocker and disopyramide has not shown excessive ejection fraction reduction and has demonstrated acceptable safety [35]. However, the global strain assessed by 2D-STE was reduced by disopyramide in humans with HOCM [36]. In this study, despite using a combination of carvedilol and disopyramide, no myocardial dysfunction based on LV myocardial strain was observed in cats. Therefore, adding disopyramide could be safe without causing excessive myocardial dysfunction in cats with HOCM. However, this study examined the efficacy of disopyramide only in cases of early-stage HOCM. Further studies are needed to verify the safety of disopyramide in cats with more progressed HOCM.

In cats, myocardial function has been reported to improve as a result of LVOTO alleviation by β-blockers [32]. However, no improvement was observed in either longitudinal or circumferential strain after disopyramide administration in this study. As a hypothesis, successful LVOTO alleviation with the combination of carvedilol and disopyramide is presumed to improve myocardial function, but this improvement is inferred to be offset by the negative inotropic effects of both drugs.

The present study observed a reduction in IVSd following the addition of disopyramide. Previous reports in cats with HOCM where carvedilol monotherapy successfully alleviated LVOTO have also noted an improvement in LV wall thickness (IVSd and LVPWd) [32]. Similarly, in humans, LVOTO relief has led to anatomical improvements [37]. Therefore, the disopyramide-induced relief of LVOTO in this study might contribute to reducing LV wall stress, and lead to wall thickness reduction. Furthermore, cats with HOCM non-responsive to carvedilol have been reported to exhibit significantly thicker IVSd and longer mitral valve anterior leaflets compared to those of the responder group [10]. Therefore, the anatomical improvements in the LV observed in this study is suggested to further contribute to the alleviation of LVOTO.

In this study, before disopyramide treatment, LVOTV_rest_ was below 3.5 m/s in four of six cats, while LVOTV_excited_ was 3.5 m/s or higher in all cats. In humans, when the LVOT pressure gradient at rest is <30 mmHg, evaluation of LVOTV with exercise stress test is recommended due to the variability of LVOTV. LVOTV can be influenced by various factors in humans: heart rate, blood pressure, volume status, activity, medications, food, and alcohol intake [4,38,39]. However, no consensus guidelines exist for LVOTV assessment in cats. Given that cats are sympathetically dominant, the inherent stress of examination may also influence LVOTV in cats. Considering these points, the significant reduction in LVOTV_excited_ observed after the addition of disopyramide is considered a clinically valuable finding.

In this study, cTnI levels significantly decreased following disopyramide administration. cTnI is a biomarker reflecting myocardial cell injury, and its utility for monitoring treatment in humans with HOCM has been reported [40]. Furthermore, elevated cTnI is considered a poor prognostic factor in cats with HCM [41,42]. This study also found a significant positive correlation between cTnI and LVOTV. These findings suggest that the observed reduction in cTnI concentration is likely a result of LVOT relief with disopyramide, potentially enhancing the necessity of LVOTV treatment in cats.

Several side effects may be of concern when administering disopyramide, associated with prolongation of the QT interval and anticholinergic effects [14]. However, no QT interval prolongation was observed following disopyramide administration. In humans, due to concerns of potentially fatal arrhythmias such as Torsade de Pointes associated with QT interval prolongation by disopyramide, ECG monitoring is typically performed [43]. Furthermore, a previous report noted only a slight QT prolongation, which did not necessitate discontinuation of disopyramide [14]. Additionally, no anticholinergic side effects such as tachycardia, constipation, or dysuria were observed. Therefore, our findings suggest that the administration protocol used in this study can be tolerable for cats with HOCM. However, one cat withdrew from the study due to poor medication compliance caused by its taste and although not observed in this study, it is important to consider the potential for appetite loss resulting from decreased gastrointestinal peristalsis due to vagal nerve blockade. Therefore, if gastrointestinal symptoms occur due to disopyramide, it may be necessary to adjust the medication dosage.

This study had several limitations. First, he small sample size might have affected the results. In addition, the incidence of adverse effects may have changed with the accumulation of more cases in future studies. Second, this study investigated the efficacy of disopyramide only at a single dose and frequency. Since a shorter half-life has been reported in dogs (necessitating q 8h dosing), an appropriate method of administration was necessary. Further research is needed to establish an ideal dosage of disopyramide for cats with HOCM. Third, bias may have occurred due to the selected study group. As the results in this study are exclusively from cats in ACVIM Stage B1, they could not be directly extrapolated to HOCM cats in other stages. Furthermore, there may be cases with HOCM for which disopyramide is not effective. Although all cats in this study responded to the addition of disopyramide, some humans do not respond even to combination therapy with disopyramide and beta-blockers and ultimately require surgical intervention [4,5]. Therefore, similar cases that were non-responsive to disopyramide may also emerge in cats.

## 5. Conclusions

In cats with HOCM non-responsive to carvedilol treatment, the concurrent administration of disopyramide significantly reduced LVOTO. This improvement was accompanied by a significant reduction in cTnI levels. Furthermore, anticipated adverse effects, such as worsening of myocardial function due to negative inotropic action, prolonged QT intervals, and anticholinergic effects, were not observed. These findings suggest that the combination of carvedilol and disopyramide may offer a valuable therapeutic option for managing cats with HOCM non-responsive to carvedilol treatment.

## Figures and Tables

**Table 1:** Results of clinical characteristics for Pre and Post disopyramide treatment in cats with obstructive hypertrophic cardiomyopathy.

Variables	Pre (*n* = 6)	Post (*n* = 6)	*p* Value
Nunber (Male/Female)	6 (3/3)		
Age (y)	2.1 (1.1, 3.7)	2.2 (1.4, 3.9)	0.055
Body weight (kg)	3.3 (3.0, 4.5)	3.3 (3.0, 4.4)	0.673
Systolic blood pressure (mmHg)	133 (124, 139)	125 (119, 131)	0.137
HR (bpm)	174 (156, 188)	180 (171, 189)	0.513
QT (ms)	186 (166, 208)	186 (168, 206)	0.653
QTc (ms)	34.3 (28.6, 38.6)	33.5 (29.1, 37.3)	0.851
cTnI (ng/mL)	0.102 (0.035, 0.248)	0.047 (0.003, 0.135)	0.038
Carvedilol dose (mg/kg q 12h)	0.6 (0.6, 0.6)	0.6 (0.5, 0.6)	
Disopyramide dose (mg/kg q 12h)		11.4 (8.5, 12.5)	
Duration of disopyramide (days)		56 (19, 138)	

**Table 2:** Results of echocardiographic variables for Pre and Post disopyramide treatment in cats with obstructive hypertrophic cardiomyopathy.

Variables	Pre (*n* = 6)	Post (*n* = 6)	*p* Value
LA/Ao	1.2 (1.1, 1.3)	1.0 (0.9, 1.4)	0.334
IVSd (mm)	5.5 (5.2, 6.5)	5.3 (4.5, 6.1)	0.028
LVIDd (mm)	12.1 (11.6, 13.0)	12.9 (11.1, 13.9)	0.642
LVPWd (mm)	5.8 (5.4, 5.9)	5.1 (4.5, 5.7)	0.293
RWT	0.9 (0.8, 1.0)	0.7 (0.5, 0.7)	0.518
FS (%)	40.1 (34.5, 43.8)	44.0 (38.4, 52.4)	0.464
E-wave (m/s)	0.7 (0.6, 0.8)	0.7 (0.5, 0.7)	0.462
E/A	1.1 (0.6, 0.8) *n* = 5	1.0 (0.9, 1.0) *n* = 5	0.144
LVOTV_rest_ (m/s)	3.0 (2.5, 4.9)	1.2 (0.9, 1.6) *	0.003
LVOTV_excited_ (m/s)	4.5 (4.1, 4.9)	2.0 (1.3, 2.7) *	<0.001

*: The value was significantly different between Pre and Post groups (*p* < 0.05). FS: fractional shortening; IVSd: end-diastolic interventricular septum thickness; LA/Ao: left atrial-to-aortic ratio; LV: left ventricular; LVIDd: end-diastolic LV internal dimension; LVIDs: end-systolic LV internal dimension; LVOTV: LV outflow tract velocity; LVPWd: end-diastolic LV posterior wall thickness; RWT: relative wall thickness.

**Table 3:** Results of two-dimensional speckle-tracking echocardiography variables for Pre and Post disopyramide treatment in cats with obstructive hypertrophic cardiomyopathy.

Variables	Pre (*n* = 6)	Post (*n* = 6)	*p* Value
Longitudinal strains (%)			
Whole layer	16.2 (13.4, 18.0)	10.0 (9.2, 19.0)	0.345
Endocardial layer	19.2 (14.3, 22.9)	11.2 (10.3, 22.2)	0.345
Epicardial layer	13.3 (11.2, 14.4)	9.1 (8.3, 16.5)	0.753
Endo/Epi	1.5 (1.1, 1.6)	1.3 (1.2, 1.3)	0.556
Circumferential strains (%)			
Whole layer	16.0 (15.4, 20.6)	19.9 (16.1, 20.8)	0.380
Endocardial layer	32.9 (28.2, 36.9)	36.2 (27.2, 39.3)	0.752
Epicardial layer	8.0 (5.5, 8.8)	8.3 (7.9, 8.9)	0.249
Endo/Epi	4.5 (3.5, 6.0)	3.9 (3.5, 4.7)	0.265

Endo/Epi: endocardial to epicardial strain ratio.

**Table 4:** Correlations among cardiac troponin I and left ventricular outflow tract velocity and myocardial strains.

Variables	cTnI		LVOTV_rest_		LVOTV_excited_	
	*r*	*p* Value	*r*	*p* Value	*r*	*p* Value
cTnI	―	―	0.62	<0.01	0.66	0.020
Longitudinal strains (%)						
Whole layer	−0.53	0.074	−0.03	0.936	−0.01	0.985
Endocardial layer	−0.52	0.081	0.01	0.970	0.01	0.980
Epicardial layer	−0.45	0.143	−0.07	0.836	−0.08	0.808
Endo/Epi	−0.33	0.301	0.19	0.552	0.12	0.719
Circumferential strains (%)						
Whole layer	−0.20	0.541	−0.11	0.743	−0.14	0.669
Endocardial layer	−0.25	0.428	0.03	0.926	−0.03	0.934
Epicardial layer	−0.06	0.862	−0.20	0.540	−0.18	0.571
Endo/Epi	−0.04	0.914	0.22	0.499	0.16	0.617

The *r* represents Pearson’s correlation coefficient for normally distributed data or Spearman’s correlation coefficient for non-normally distributed data. cTnI: cardiac troponin I; Endo/Epi: endocardial to epicardial strain ratio; LVOTV: LV outflow tract velocity.

## Data Availability

The datasets used or analyzed in the current study are available from the corresponding author upon reasonable request.
